# Change in Osmotic Pressure Influences the Absorption Spectrum of Hemoglobin inside Red Blood Cells

**DOI:** 10.3390/cells13070589

**Published:** 2024-03-28

**Authors:** Miroslav Karabaliev, Bilyana Tacheva, Boyana Paarvanova, Radostina Georgieva

**Affiliations:** 1 Department of Physics and Biophysics, Faculty of Medicine, Trakia University, 11 Armeiska, 6000 Stara Zagora, Bulgaria; bilyana.tacheva@trakia-uni.bg (B.T.); boyana.parvanova@trakia-uni.bg (B.P.); 2 Institute of Transfusion Medicine, Charite-Universitatsrnedizin Berlin, Chariteplatz 1, 10117 Berlin, Germany

**Keywords:** red blood cells, osmosis, hemoglobin, absorption spectrum, Soret peak

## Abstract

Absorption spectra of red blood cell (RBC) suspensions are investigated in an osmolarity range in the medium from 200 mOsm to 900 mOsm. Three spectral parameters are used to characterize the process of swelling or shrinkage of RBC—the absorbance at 700 nm, the Soret peak height relative to the spectrum background, and the Soret peak wavelength. We show that with an increase in the osmolarity, the absorbance at 700 nm increases and the Soret peak relative height decreases. These changes are related to the changes in the RBC volume and the resulting increase in the hemoglobin intracellular concentration and index of refraction. Confocal microscopy and flow cytometry measurements supported these conclusions. The maximum wavelength of the Soret peak increases with increasing osmolarity due to changes in the oxygenation state of hemoglobin. Using these spectrum parameters, the process of osmosis in RBCs can be followed in real time, but it can also be applied to various processes, leading to changes in the volume and shape of RBCs. Therefore, we conclude that UV–Vis absorption spectrophotometry offers a convenient, easily accessible, and cost-effective method to monitor changes in RBC, which can find applications in the field of drug discovery and diagnostics of RBC and hemoglobin disorders.

## 1. Introduction

UV–Vis spectroscopy is a method that is often used in modern scientific laboratories due to its high accessibility. Spectrophotometers have applications in many research areas and represent basic equipment in almost every laboratory. Spectrophotometry is a widely applied method in physics, materials science, chemistry, biochemistry, molecular biology, pharmacology, and medicine, etc., mainly for the qualitative and quantitative analysis of the concentration or composition of solutions and dispersed systems [1,2,3].

In medical diagnostics, UV–Vis—spectrophotometry is a sensitive and routinely used method for studying physical, chemical, and physiological changes in the composition of blood and also in the shape and structure of the blood cells [4,5,6].

Erythrocytes, also known as red blood cells (RBCs), constitute the largest cell population in the blood, with a concentration of 45% by volume and roughly 5.5 × 10^6^ cells/μL [7,8]. For comparison, white blood cells (leucocytes) occupy only 1% of the blood volume. RBCs are responsible for oxygen and carbon dioxide transport between the lungs and tissues in the body. This function is performed by a single type of protein, hemoglobin, which makes up 95% of the protein content of the RBCs. Some of the most important diagnostic measurements are the determination of hemoglobin concentrations in the blood and its oxygenation state. Additionally, the amount of oxidized hemoglobin (methemoglobin) is measured. These parameters are obtained by a single spectrophotometric measurement of the light absorption at specific wavelengths that correspond to characteristic absorption maxima of oxy- and de-oxy-hemoglobin as well as for methemoglobin. The oxy-hemoglobin (HbO_2_) has absorption maximums at 415 nm (Soret’s band) and at 542 nm and 578 nm (Q-bands); deoxy-hemoglobin absorbs at 430 nm and 560 nm; and methemoglobin has a Soret’s band at 405 nm and 603 nm [6]. Commonly, RBCs are previously destroyed to obtain a clear hemoglobin solution, avoiding the scattering typically occurring in dispersed systems like cell suspensions.

On the other hand, scattering measurements can provide additional and highly valuable information about the integrity, size, and shape of the structural elements in the blood and specifically about these parameters of the RBCs. Light scattering is the main factor that determines the optical properties of dispersed systems. Scattering mechanisms are determined by the size of the scattering particles (L) and the wavelength (λ) of the light [9]. The scattering by small particles (bio-macromolecules, liposomes, viruses, etc.) smaller than the light wavelength (L/λ << 1) is in all directions—so-called wide-angle scattering is observed. In this case, the intensity of the scattered light depends inversely on the wavelength to the power of four. Scattering by larger particles L/λ >>1 (bacteria, cells, dust particles, etc.) occurs by a different mechanism. The light beam is reflected and refracted many times by the particles, and the scattered light is mainly detected forward—small-angle scattering [10]. The optical properties of these systems will be determined by the type of solvent, concentrations of the different compounds, size, and refractive index of the particles, as well as the refractive index of the medium. In the case when spectrophotometry is used in the investigation of whole blood or RBS suspensions, the light attenuation will be a result of both scattering and absorption by each individual particle (RBC).

The scattering depends basically on the difference in refractive indices of the suspension medium and the particle, as well as on the particle size. Therefore, any changes in the refractive index and/or size of the RBCs will cause changes in the intensity of the transmitted light.

RBCs are known for their ability to react to changes in different factors of the environment by changing their volume and shape. At physiological conditions, the volume of RBC is about 90 µm^3^, and the refractive index is 1.400. The volume can vary from about 72 µm^3^ in hypertonic to about 150 µm^3^ in hypotonic conditions. These changes in erythrocyte volume, and at a constant hemoglobin volume of 23 µm^3^, also lead to a change in the refractive index of the RBCs and in the intensity of the scattered light [11,12].

There are many pathological conditions that lead to changes in the volume and shape of erythrocytes. Some of them are because of abnormal changes in the osmotic pressure of the blood plasma. They can be divided into two groups—hyperosmolar, such as Hyperosmolar Hyperglycemic Syndrome [13], and hypoosmolar—Syndrome of Inadequate Secretion of Antidiuretic Hormone (SIADH) [14]. Blood cells would be in similar non-isotonic conditions in other situations related to dehydration of the body, transfusion of fluids and drugs, injection of contrast agents into the bloodstream for imaging, etc. There are many hereditary anemias that are associated with disorders in the volume of erythrocytes. They are a result of changes in cellular hydration due to abnormalities in membrane ion permeability [15]. Conditions with increased hydration of erythrocytes are classified as Overhydrated Stomatocytosis OHSt (or hereditary hydrocytosis). In this hemolytic anemia, there are large numbers of stomatocytes in the blood circulation. OHSt is associated with increased sodium permeability of the erythrocyte membranes [16] due to band 3 deficiency or mutations. The result is a significant increase in the volume of erythrocytes to 100–150 fL due to the increase in the concentration of sodium ions and the simultaneous entry of water into the cytoplasm. Cryohydrocytosis is an OHSt variant in which ion leakage is stimulated by low temperatures [17]. Other disorders associated with erythrocyte dehydration and reduced cell volume include sickle cell anemia, thalassemia, hereditary spherocytosis, and erythrocyte dehydration associated with malarial invasion [18]. In most of these conditions, there is an excessive leakage of potassium ions, causing dehydration of the erythrocytes and an increased hemoglobin concentration [16].

Additionally, volume and shape changes in the RBC caused by different osmotic conditions can influence their stability against compounds with hemolytic activity, including different drugs like antibiotics, cytostatics, lectins, etc. In a previous study, we investigated the influence of osmotic pressure on the stability of RBCs in the presence of saponin. Saponins belong to the group of detergents and act as hemolytic agents. We found that the osmotic pressure of the suspension medium has a strong influence on the critical concentration of saponin at which hemolysis appears. Interestingly, these concentrations are lower at hypertonic conditions, and in real-time measurements, hemolysis is accelerated at hypertonicity compared to iso- and hypotonicity for equal saponin concentrations [19]. On the other hand, long-term exposure to some species that do not cause hemolysis directly, like glucose, can influence the fragility of RBCs under hypoosmotic conditions. This was shown in [20] for RBCs exposed to high concentrations of glucose (35 mM) in vitro, as well as for the RBCs of diabetic patients.

While the influence of volume size and shape of RBCs during osmotic changes has been well studied, the oxygenation state of hemoglobin during these processes has not received much attention. There are some investigations that focus on the spectral changes in hemoglobin during storage [21], increased or decreased oxygen partial pressure [22], or the presence of some organic molecules [23]. However, with increasing hemoglobin concentration in the cells during osmotic shrinking, the accessibility of the binding site for oxygen (the hems) could decrease and cause some changes in the spectrum of hemoglobin-like shifts of the maxima in the specific wavelength ranges.

In this study, we investigated the optical spectra of intact native human RBCs undergoing osmotic swelling or shrinking in the range of 250 to 750 nm. Our attention was focused on the scattering behavior of the suspensions during specific size and shape transitions of the RBCs and on the analysis of the height and position of the Soret peak with respect to possible changes in the oxygen saturation of hemoglobin in the cells.

## 2. Materials and Methods

### 2.1. RBC Suspension Sample Preparation

RBC suspension samples were prepared from venous blood that was taken from healthy volunteer donors on the day of investigation in the clinical laboratory of the University hospital of Trakia University, Stara Zagora, Bulgaria, and conducted with the informed consent of patients in accordance with the Declaration of Helsinki and the protocol No 10/5 June 2019 of Ethic Commission of the Medical Faculty, Trakia University, Stara Zagora, Bulgaria.

Blood was centrifuged at 1700× *g* for 3 min and RBCs were separated from blood plasma. Isolated erythrocytes were washed two times in 150 mM NaCl and packed to a hematocrit of about 95%. The initial suspension used in the experiments was with hematocrit (Hct) 10% in saline (150 mM NaCl). From this suspension, small portions of 20 µL were taken and dissolved in 3 mL NaCl solutions with various osmolarities ranging from 200 mOsm to 900 mOsm. Sodium chloride (Sigma, USA) was used to prepare 100 mM, 150 mM, 225 mM, 300 mM, 375 mM, and 450 mM suspension media with corresponding osmolarities 200 mOsm, 300 mOsm, 450 mOsm, 600 mOsm, 750 mOsm, and 900 mOsm.

### 2.2. UV–Vis Absorption Spectra Measurements

The RBC suspension samples were characterized by measuring their UV–Vis absorption spectrum. The suspension with an initial hematocrit of 0.067% was prepared directly in a measuring quartz cuvette with a volume of 3 mL and an optical pathlength of 1 cm. After the addition of RBCs to the medium prefilled in the cuvette, the suspension was stirred, and the cuvette was put in the spectrophotometer. The RBCs started to swallow, shrink, or remain the same depending on the tonicity of the medium. During this process, the absorption spectrum of the RBC suspension was measured every 15 s in the wavelength range of 300 nm to 750 nm at intervals of 0.5 nm. The spectrophotometer used for the experiments was Cary 60 UV–Vis (Agilent Technologies, Santa Clara, CA, USA).

Due to the osmosis and the changes in the RBC volume, the spectra changed until a steady state was reached after several minutes. At the end of the process, Triton X-100 was added to the suspension in order to hemolyze the RBCs, and the spectrum of the released hemoglobin was measured. Triton X-100 was purchased from Merck, Germany, and sodium chloride was purchased from Sigma-ALDRICH, St. Louis, MO, USA.

### 2.3. Absorption Spectra Processing

Changes in the absorption spectrum were observed until the RBC shape and volume reached equilibrium at the given osmolarity. In order to follow the process throughout the course of these changes, several parameters of the spectra were monitored.

The measured absorbance at different wavelengths is the result of the scattering of light from intact RBCs and absorption from hemoglobin. Scattering causes a decrease in the intensity of the light transmitted through the cuvette and, hence, an increase in the measured absorbance, which is not a result of real light absorption. For this reason, we denoted the measured absorbance as apparent absorbance.

The first monitored parameter is the measured absorbance at 700 nm (A700). At this wavelength, the Hb does not absorb light, so the apparent absorbance is only due to scattering.

The second parameter is the hemoglobin Soret peak height. In suspension, the absolute value of the absorbance of the Soret peak is the sum of the Hb absorption and the spectrum background due to the scattering. For this reason, we used the relative peak height of the Soret peak as a characterization parameter, which is the difference between the measured absorbance at the peak wavelength and the measured absorbance at a wavelength of 500 nm [24].

The third monitored parameter is the Soret peak wavelength. Because the resolution used to measure the spectrum was 0.5 nm, in order to determine more precisely the wavelength of the peak, we used a procedure described in detail previously in [25]. In brief, the procedure consists of calculating the first derivative of the spectrum and evaluating the exact wavelength at which the first derivative has zero value in the range of the Soret peak and, correspondingly, the spectrum has a maximum.

### 2.4. Confocal Laser Scanning Microscopy (CLSM)

RBCs were imaged using a confocal laser scanning microscope (LSM 510 Meta, Carl Zeiss AG, Oberkochen, Germany) with a 100× oil-immersion objective (n.a. 1.3). RBC suspensions were prepared in phosphate-buffered NaCl solutions at a pH of 7.4 with osmolarities of 200, 300, 600, and 900 mOsm, respectively. Each solution was additionally supplemented with 2% human serum albumin (HSA) for shape stabilization. A 20 μL drop of each suspension was placed on a cover slip. The images were obtained at 488 nm (Argon laser) in the transmission mode.

### 2.5. Flow Cytometry

The scattering behavior of RBCs at 200, 300, 600, and 900 mOsm was studied using a flow cytometer (BD FACS Canto II, Franklin Lakes, NJ, USA). The cells were identified by the forward scatter (FSC) and the sideward scatter (SSC) and were displayed as a contour diagram. The scattering intensity distribution in both channels was displayed in corresponding diagrams. The data analysis was performed by the FACS-Diva software v7.0.

## 3. Results and Discussion

**Figure 1:**
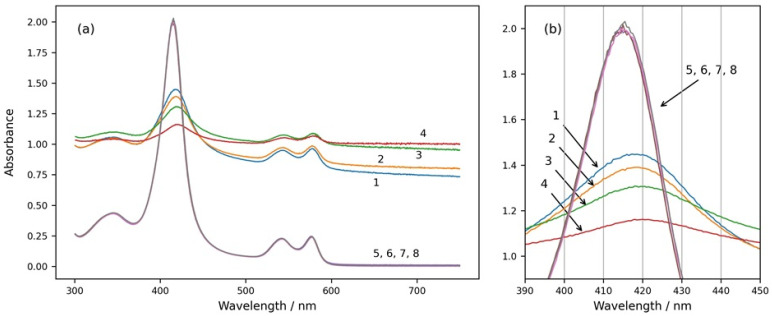
(**a**) Absorption spectra of intact and hemolyzed RBCs in suspension media with different osmolarities—200 mOsm NaCl (curves 1 and 5), 300 mOsm NaCl (curves 2 and 6), 600 mOsm NaCl (curves 3 and 7), and 900 mOsm NaCl (curves 4 and 8). Curves 1, 2, 3, and 4 are spectra of suspensions with intact erythrocytes. Curves 5, 6, 7, and 8 are obtained after the same suspensions were treated with Triton X-100 and the RBCs were hemolyzed. (**b**) The same graph enlarged in the wavelength range of the Soret peak from 390 nm to 450 nm. Initial hemat.ocrit 0.067%.

Four spectra of suspensions with the same number of erythrocytes dissolved in media with different osmolarities are shown in Figure 1 (spectra 1, 2, 3, and 4). The spectra were taken 15 min after the suspension preparation, when there was no more swelling or shrinkage of RBCs and a steady state was reached. The spectra 5, 6, 7, and 8 shown in the same graph were obtained after the same suspensions were treated with Triton X-100 and the erythrocytes were completely hemolyzed.

From Figure 1, it can be seen that the spectra of intact RBCs have high background apparent absorption for all wavelengths due to the light scattering from the cells (spectra 1, 2, 3, and 4). In contrast, the spectra of solutions with hemolyzed cells do not have background absorption because of the lack of scattering, and they do not change with the osmolarity of the medium (spectra 5, 6, 7, and 8). The absolute height as well as the relative height of the Soret peak of RBC suspension spectra are smaller than those of the Soret peaks of solutions with hemolyzed RBCs, and they decrease with increasing osmolarity of the suspension medium. The Soret peaks of RBC suspension spectra are shifted to higher wavelengths in comparison to the Soret peaks wavelengths of the spectra after hemolysis of the RBCs, and additionally, the wavelengths at the maximum of the Soret peak of RBC suspensions depend on the osmolarity of the medium and increase with increasing osmolarity.

As is well known and established, scattering is due to the different indexes of refraction of the medium and the dispersed particles, which in our case are intact RBCs. The main factors that influence scattering are also the size and shape of the particles [11].

**Figure 2:**
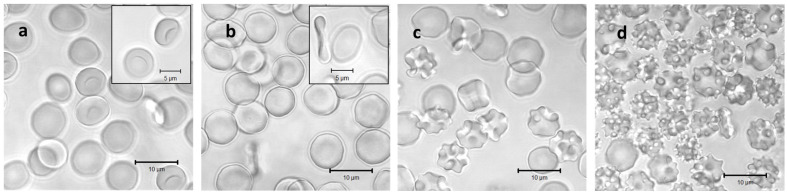
Confocal laser scanning images of washed RBCs incubated in solutions with osmotic pressures of 200 mOsm (**a**), 300 mOsm (**b**), 600 mOsm (**c**), and 900 mOsm (**d**), respectively. Each solution was additionally supplemented with 2% human serum albumin for shape stabilization during microscopic analysis. Inserts in (**a**,**b**) highlight the stomatocyte and discocyte shapes at the hypotonic and the isotonic conditions, respectively.

RBCs at different osmolarities were also observed by confocal microscopy to visualize their shape and volume (Figure 2). As can be seen from the confocal micrographs in Figure 2, the RBCs undergo significant shape transitions, being stomatocytes at hypotonic 200 mOsm, discocytes at isotonic 300 mOsm, and echinocytes at hypertonic 600 and 900 mOsm. Simultaneously, their volume and dimensions decrease in the same order due to osmotic swelling at 200 mOsm and shrinking at 600 and 900 mOsm. These changes are reflected by the apparent background absorption in spectra 1 to 4 of Figure 1. 

**Figure 3:**
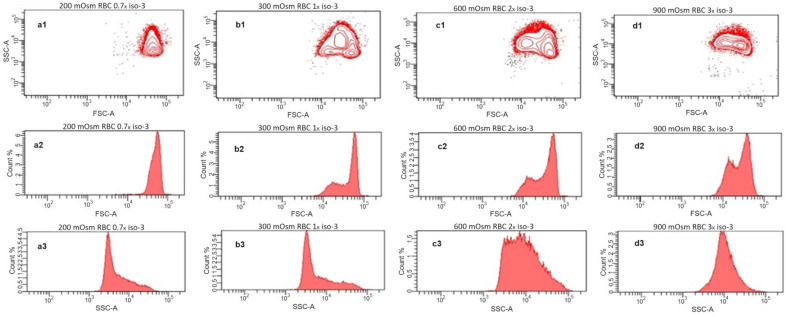
Flow cytometry analysis of washed RBCs incubated in solutions with osmotic pressures of 200 mOsm (**a1**–**a3**), 300 mOsm (**b1**–**b3**), 600 mOsm (**c1**–**c3**), and 900 mOsm (**d1**–**d3**). The upper row (**a1**–**d1**) shows site scatter (SSC) vs. forward scatter (FSC) contour diagrams; the middle row (**a2**–**d2**) represents the corresponding histograms of the detected FSC intensities; and the lower row (**a3**–**d3**) depicts the histograms of the SSC intensities.

Further, we investigated the scattering behavior of the RBC suspensions at different osmolarities by flow cytometry (Figure 3). Here, the single cells are detected and characterized by their forward and side scattering (contour diagrams Figure 3(a1–d1)), and the distributions of scattering intensities of each sample are presented as histograms for the forward (Figure 3(a2–d2)) and side (Figure 3(a3–d3)) scatters, respectively. Generally, the forward scatter is directly related to the size of the detected particles. The side scatter intensity reflects the complexity of the shape and structure of the particles. On the contour diagram of the sample at hypoosmotic conditions (200 mOsm), RBCs form a single, uniform population (Figure 3(a1)) with a narrow distribution of the forward scattering intensity (Figure 3(a2)) and a relatively low maximum of side scattering. At these conditions, RBCs are swollen and closer to the spherical shape, which diminishes the influence of orientation on both the forward and the side scattering properties. In the sample at physiological osmolarity (isotonicity, 300 mOsm), the population appears inhomogeneous due to the disc-like shape and the strong dependence of the scattering on the orientation of the cells relative to the direction of the laser beam (Figure 3(b1)). This is more obvious in the intensity distribution of the forward scatter (Figure 3(b2)), where an additional maximum at lower intensities appears, reflecting the smaller thickness of the RBCs with a discocytic shape. Such bimodal distribution in the forward scattering was already reported for RBCs at physiological conditions [26,27,28]. In the distribution of the side scatter intensities (Figure 3(b3)), only a slight increase toward higher scattering intensities is observed. The inhomogeneous appearance of the RBC population remains for the samples at the hypertonic 600 mOsm (Figure 3(c1)) and 900 mOsm (Figure 3(d1)). Here, the water loss leads to shrinkage and flattening of the RBCs and a transition to the irregular echinocytic shape. The increasing flattening leads to a more pronounced bimodality in the intensity distribution of the forward scattering with increasing hypertonicity (Figure 3(c2,d2)). The side scattering moves toward higher intensities, finally forming a broad distribution at 600 mOsm (Figure 3(c3)) and a homogeneous distribution only with a maximum at 900 mOsm (Figure 3(d3)). The increased site scattering can be explained by the irregular shape on the one hand, but also by the increased values of the refractive index of the RBCs caused by increasing intracellular hemoglobin concentrations during shrinkage on the other hand [29].

**Figure 4:**
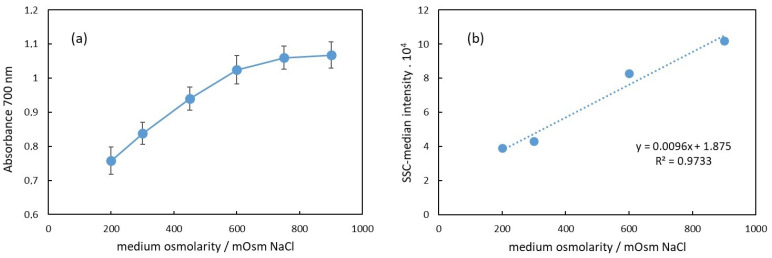
(**a**) Dependence of the apparent absorbance at 700 nm of RBC suspension on the suspension medium osmolarity. (**b**) Median of the side scattering intensity obtained in flow cytometry as dependence on the medium osmolarity.

Figure 4a shows the dependence of the apparent absorbance at 700 nm on the osmolarity of the medium of RBC suspensions. This wavelength was chosen because the hemoglobin does not absorb light at 700 nm; hence, the apparent absorption is due to scattering and, correspondingly, to decreased transmittance of the light. As is well known and established, scattering is due to different indexes of refraction of the medium and the dispersed particles, which in our case are the intact erythrocytes [11]. It has also been shown that the scattering properties of blood depend on the erythrocyte volume, shape, and hematocrit [29]. In Figure 4b, the median side scattering intensity as obtained from the flow cytometry data is displayed in dependence on the medium osmolarity of the RBC suspension. Compared to the results shown in Figure 4a,b, we can conclude that the increase in the apparent absorbance with the osmolarity is directly related to the increased side scattering.

In Figure 4a, the apparent absorbance increases with the osmolarity in the range from 200 mOsm to 900 mOsm NaCl. Specifically, in the 200–600 mOsm range, the dependence appears to be linear. This change in absorbance at 700 nm correlates with alterations in the erythrocyte volume. 

The erythrocyte volume varies with osmolarity due to water flux in or out of the cell, driven by osmotic pressure differences between the cell and the surrounding medium. These volume changes affect the erythrocyte’s relative refractive index, which is primarily influenced by the protein concentration inside the cells. Under hypotonic conditions (200 mOsm), erythrocytes swell due to water influx, resulting in a lower protein concentration than that in a physiological medium (300 mOsm). Consequently, the relative index of refraction decreases, leading to reduced scattering and a lower apparent absorbance of the suspension, as observed in Figure 4a. In hypertonic conditions, the opposite occurs. Above 300 mOsm, erythrocytes begin to shrink due to water outflow, increasing the protein concentration and refractive index. This results in enhanced scattering and increased apparent background absorbance. 

These findings are in corroboration with the results published by other authors. Mazeron et al. [11] found that during the swelling of erythrocytes in hypotonic media, the intensity of scattered light forward (small-angle scattering) initially increases due to an increase in the volume of erythrocytes. As a result, their refractive index decreases, and the forward scattering decreases. They measured the extinction during the shrinking and swelling of erythrocytes and found that if the angle of detection of the transmitted light is large enough, the shrinkage leads to a decrease and the swelling to an increase in the light transmittance of the suspension.

In our results, the linear dependence of the absorbance in the range 200–600 mOsm suggests that the scattering is “proportional” to the protein concentration or density inside the cell. The volume of the cell in different osmotic conditions is inversely proportional to the osmolarity of the medium [30,31]. Since during osmosis, the total protein amount in the cell does not change, the protein concentration inside the cell is inversely proportional to the volume of the cell and, hence, proportional to the osmolarity of the medium. Thus, we can assume that the index of refraction of RBC increases linearly with the increase in osmolarity. Other authors found that the refractive index of human deoxygenated and oxygenated hemoglobin between 400 and 700 nm depended linearly on the hemoglobin concentration for hemoglobin concentrations up to 140 g/L [32]. Paul et al. [33] experimentally determined the average total Hb mass and volume for single RBCs that have a discocyte, echinocyte, and spherocyte shape. The results show that there is a difference in erythrocyte density depending on the shape. Spherocytes have the smallest density, and echinocytes have the highest [33]. The difference in the RBC density suggests a difference in the refractive indexes of light for cells with different volumes.

Our results presented in Figure 4a are in agreement with this assumption in the range of osmolarity from 200 mOsm to 600 mOsm, where the dependence of the apparent absorbance on the osmolarity is linear. The deviation from linearity above 600 mOsm could be due to a decrease in the size of erythrocytes, which could result in decreased scattering.

**Figure 5:**
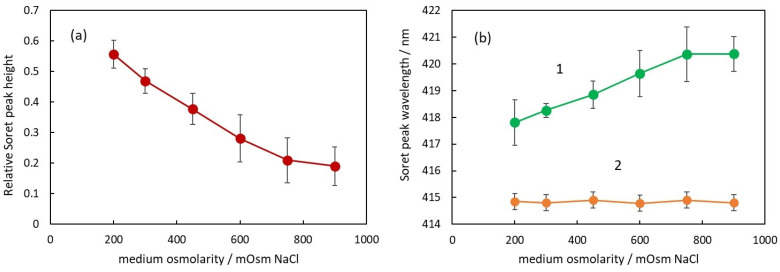
(**a**) Relative Soret peak height of RBC suspensions in media with different osmolarities. (**b**) Soret peak wavelength of suspensions with intact RBC (curve 1) and after the same suspensions were treated with Triton X-100 to hemolyze the RBC (curve 2).

The second parameter to characterize the absorption spectrum is the relative height of Soret peak. In Figure 5a, the dependence of the Soret peak relative height on the osmolarity of the medium is presented.

It can be seen that the Soret peak height decreases with the increase in osmolarity. The dependence looks linear up to 750 mOsM, and above this concentration, there is a deviation from linearity similar to the deviation observed in Figure 4a. Comparing the data in Figure 4a and Figure 5a, it can be seen that there is a negative correlation between the apparent absorbance and the Soret peak relative height, i.e., when the apparent absorbance increases with osmolarity, the Soret peak relative height decreases. The possible explanation for this phenomenon is the forward scattering of light from intact erythrocytes [24]. When a photon is scattered from an erythrocyte, it does not penetrate the internal erythrocyte content and, correspondingly, does not interact with the hemoglobin inside the cell. On the other hand, according to Mie’s theory, the scattering is mostly forward, which means that many of the scattered photons reach the detector of the spectrophotometer.

Analyzing the spectra, a very interesting parameter that depends on the osmolarity is the Soret peak wavelength (Figure 5b).

First of all, it should be pointed out that at all osmolarities, the Soret peak wavelength is larger for the suspensions (green curve 1) compared to the wavelength of the same peak after hemolysis (orange curve 2).

In Figure 5b, the Soret peak wavelength increases linearly with an increase in osmolarity up to 750 mOsm (green curve 1). The displacement from the wavelength value at physiological conditions is again related to the volume of the erythrocytes and the Hb concentration inside. It is not dependent on the NaCl concentration around the Hb since in solutions with hemolyzed erythrocytes, the Soret peak wavelength is the same for all NaCl concentrations (orange curve 2).

With increasing osmolarity, the change in the Soret peak wavelength is in the direction of larger wavelengths, which could be related to the partial deoxygenation of hemoglobin. When the number of deoxygenated Hb molecules increases, the Soret peak wavelength moves in a direction toward 430 nm, which is the wavelength of Soret peak of fully deoxygenated Hb.

These assumptions are in corroboration with the results of other authors showing that the Soret peak wavelength of fresh blood suspension at physiological conditions is about 418 nm [21,22], i.e., it is larger than the wavelength of the peak of free Hb in a solution (415 nm). This deviation of the wavelength should be related to the oxygenation state of Hb inside RBCs because, after additional saturation of the blood with oxygen, Abramczyk et al. [22] found that the Soret peak wavelength decreases toward 414.43 nm [22]. Wojdyla et al. [4] showed that the deformation of RBCs caused by optical traps results in partial deoxygenation of the hemoglobin inside the RBCs and a shift in the Soret peak toward 430 nm. Since this deformation is related to a decreased volume of RBCs, these results support our findings. Zhang et al. [34] investigated the influence of osmotic pressure on the oxygenation state of crosslinked hemoglobin. They found that the increase in osmolarity led to a decrease in the oxygen transport function of hemoglobin, suggesting that the exponentially increasing viscosity inside RBCs with increasing Hb concentrations reduces the rate of convective diffusion of Hb and oxygen.

**Figure 6:**
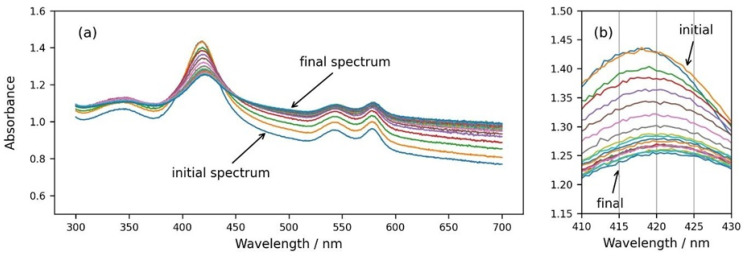
(**a**) Consecutive spectra measured every 30 s after dispersing RBCs from the physiological solution into 900 mOsm NaCl. (**b**) Same graph enlarged in the wavelength range of the Soret peak from 410 nm to 430 nm.

Finally, we followed the spectral changes during the osmotic shrinking of RBCs in real time, transferring RBCs from suspension in physiological concentration (300 mOsm) into a medium with three times higher osmolarity (900 mOsm). In Figure 6, it can be seen that immediately after the addition of the cells to a medium with higher osmolarity, the spectra start to change (Figure 6a,b), eventually reaching a stable state after a few minutes. It is obvious that the background of the RBC suspension spectrum increases and the Soret peak height decreases (Figure 6a), which is related to increased scattering due to the shrinkage of RBCs and the increase in the refractive index inside the cells. Along with the decrease in the Soret peak height, the displacement of the peak wavelength to larger values is also clearly noticeable (Figure 6b). 

**Figure 7:**
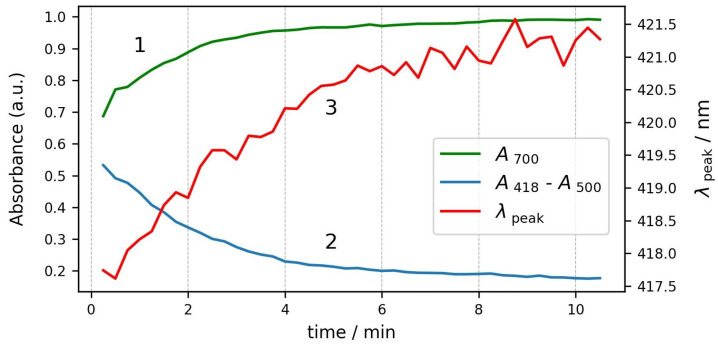
Kinetics of changes in spectrum parameters during the same process. Curve 1—absorbance at 700 nm; curve 2—Soret peak relative height; and curve 3—Soret peak wavelength.

The changes in the three parameters during the course of the process are plotted in Figure 7. All three parameters start to change simultaneously and reach stable values after about six minutes. We can conclude that the osmosis of water out of the cells is not a very fast process, and it takes several minutes until the RBCs reach a stable shape and volume.

## 4. Conclusions

In conclusion, we demonstrated that measuring the apparent absorption of RBCs in suspension is capable of delivering information about their size, shape, and oxygenation state. This is achieved by measuring the background apparent absorbance at 700 nm, the Soret peak relative height, and the Soret peak wavelength. These three above-mentioned parameters of the RBC suspension spectra could be used to monitor real-time effects caused by changes in the surrounding medium or the addition of some drugs or detergents to the RBC state. 

Generally, the method can be used to follow various processes that go with changes in the volume and shape of RBCs. In this respect, UV–Vis absorption spectrophotometry offers a convenient, easily accessible, and cost-effective method to monitor changes in the RBC volume, hemoglobin intracellular concentration, and oxygenation state, which could have numerous applications in the field of drug discovery. Here, we investigated only RBCs from healthy donors, but this method can deliver valuable information for the functionality of hemoglobin in the RBCs of diabetic patients as well as of patients with different hemoglobin disorders and anemia. The partial deoxygenation at hyperosmotic conditions that was discovered in our work could also be interesting as a hint for optimization of the conditions for storage of donated RBCs in order to achieve prolonged preservation of their stability and functionality.

## Data Availability

The data are available upon request to the authors.
